# Author Correction: Solving conformal defects in 3D conformal field theory using fuzzy sphere regularization

**DOI:** 10.1038/s41467-024-52959-2

**Published:** 2024-10-18

**Authors:** Liangdong Hu, Yin-Chen He, W. Zhu

**Affiliations:** 1 Department of Physics, School of Science, Westlake University, Hangzhou, 310030 PR China; 2 Institute of Natural Sciences, Westlake Institute for Advanced Study, 18 Shilongshan Road, Hangzhou, 310024 PR China; 3 Perimeter Institute for Theoretical Physics, Waterloo, ON N2L 2Y5 Canada

**Keywords:** Phase transitions and critical phenomena, Phase transitions and critical phenomena

Correction to: *Nature Communications* 10.1038/s41467-024-47978-y, published online 30 April 2024

The original version of this Article contained an error in Eq. 9, which was missing a factor of 2 under the square root sign on the left-hand side. Equation 9 incorrectly read:

$$\sqrt{{C}_{\hat{D}}}=\frac{2}{\pi }\frac{{\varDelta }_{O}{G}_{O}(\theta=\pi /2)}{{G}_{O\hat{D}}(\theta=\pi /2)}$$

The correct form of Eq. 9 is:

$$\sqrt{2{C}_{\hat{D}}}=\frac{2}{\pi }\frac{{\varDelta }_{O}{G}_{O}(\theta=\pi /2)}{{G}_{O\hat{D}}(\theta=\pi /2)}.$$

Additionally, two quantities computed using Eq. 9 and displayed in the last line of the Results section under Eq. 9 and in the fifth and sixth columns of Table 2 were incorrect.

The incorrect values read 0.53(3) and 0.59(18), respectively.

The correct values read 0.27(1) and 0.30(8), respectively.

This has been corrected in the PDF and HTML versions of the Article.

The derivation of Eq. 9 presented in Supplementary Note 5 of the Supplementary Information has been amended and now leads to the correct form of the equation displayed as Eq. 21 in Supplementary Note 5. The values of the two quantities computed using this equation and displayed in the last line of Supplementary Note 5 under Eq. 21 have also been corrected. In summary, the modified part of Supplementary Note 5 refers to the text between the sentence describing Eq. 17 and ending with “…bulk scalar primary operator.” and the last sentence of the Note starting with “For comparison, we calculate…”.

The previous incorrect text read:
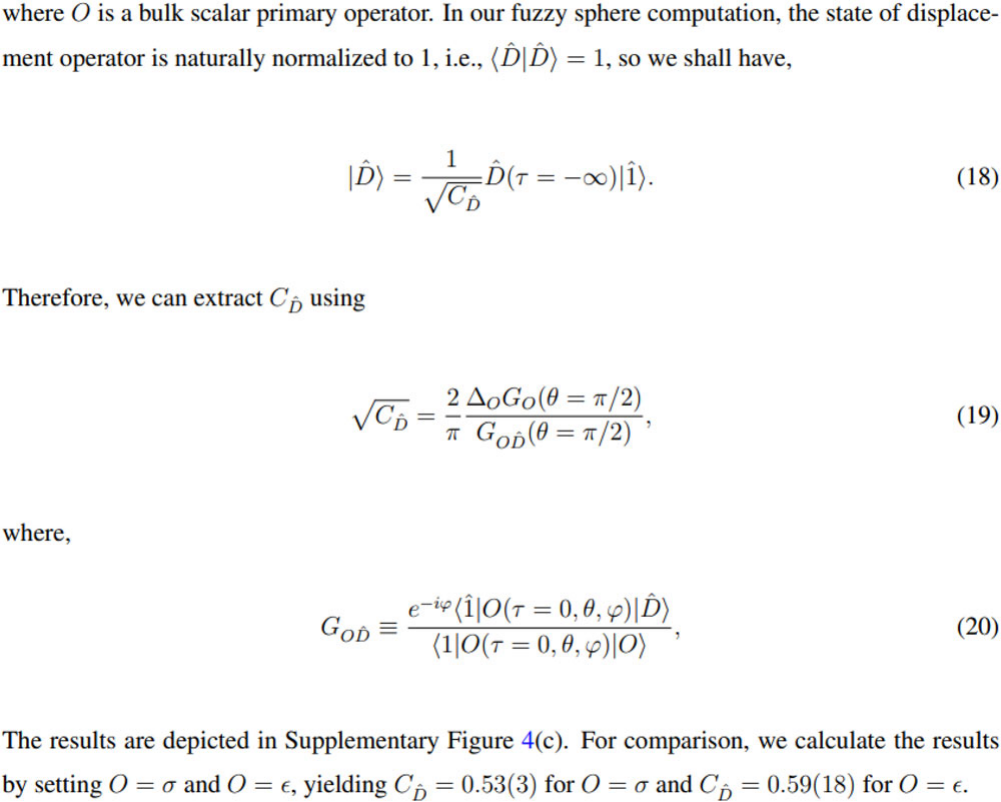


The correct text reads:
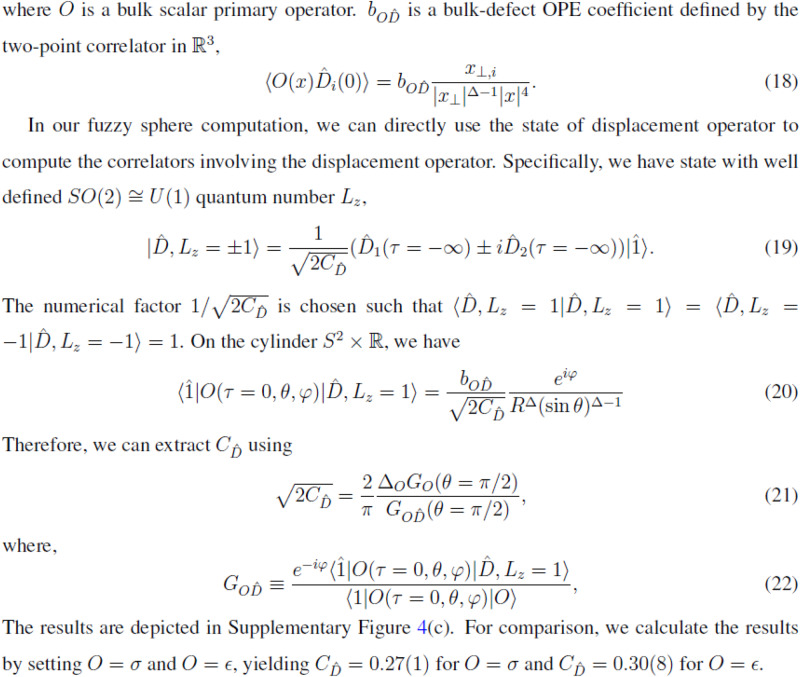


The HTML has been updated to include a corrected version of the Supplementary Information.

## Supplementary information

Revised Supplementary information

