# Supplementary material for: Solving conformal defects in 3D conformal field theory using fuzzy sphere regularization
Source: Nat Commun. 2024 Apr 30;15:3659. doi: 10.1038/s41467-024-47978-y (PMC11061275; doi:10.1038/s41467-024-47978-y)
Supplement: Supplementary file 1 — Supplementary Information [file 41467_2024_47978_MOESM1_ESM.pdf]

Supplementary Information for "Solving Conformal Defects in 3D Conformal Field Theory using Fuzzy Sphere Regularization"

Liangdong Hu

Institute of Natural Sciences, Westlake Institute for Advanced Study, 18 Shilongshan Road, Hangzhou, 310024, China and Department of Physics, School of Science, Westlake University, Hangzhou 310030, China

Yin-Chen He[*]

Perimeter Institute for Theoretical Physics, Waterloo, Ontario N2L 2Y5, Canada

W. Zhu[†]

Department of Physics, School of Science, Westlake University, Hangzhou, 310030, China

(Dated: July 21, 2024)

Supplementary Information

Supplementary information contains:

More details to support the discussion in the main text.

Fig. S1. Energy gap scaling at the critical point.

Fig. S2. Finite-size correction of scaling dimensions for the lower primaries.

Fig. S3. Correlators related to ϵ.

Fig. S4. Correlators between the displacement operator and bulk operator.

Fig .S5. Conformal tower of defect primaries in $h_d \to \infty$.

TABLE S1. The scaling dimensions of primaries in sector $L_z = 0, 1$.

TABLE S2. The scaling dimensions of primary fields under infinite and finite defect strengths.

CONTENTS

Supplementary Note 1: Bulk to defect correlators 3

Supplementary Note 2: Error analysis 4

Scaling dimension 4

OPE coefficients 6

Supplementary Note 3: Attractive fixed point 6

Supplementary Note 4: Numerical results of correlators of ϵ 8

Supplementary Note 5: Ward identity regarding to the displacement operator 9

Supplementary Note 6: In the limit of $h_d \to \infty$ 10

Supplementary References 12

In this supplementary material, we will show more details to support the discussion in the main text. In Supplementary Note 1, we discuss the defect CFT correlators on the cylinder $S^2 \times \mathbb{R}$. In Supplementary Note 2, we present an error analysis of the scaling dimensions of defect primaries. In Supplementary Note 3, we provide in-depth analysis of the attractive fixed point induced by the line defect. In Supplementary Note 4, we show the operator product expansion (OPE) related to the primary ϵ. In Supplementary Note 5 we present the computation of Zamolodchikov norm regarding to the displacement operator. In Supplementary Note 6, we discuss the physics of defect CFT in the limit of $h_d \to \infty$.

Supplementary Note 1: Bulk to defect correlators

In this section, we present the correlators of primary operators in the dCFT on the cylinder $S^2 \times \mathbb{R}$ by using the state-operator correspondence. As discussed in the main text, by introducing of a flat p-dimensional defect breaks the global conformal symmetry $SO(4,1)$ into $SO(p+1,1) \times SO(3-p)$. Making use of state-operator correspondence, the bulk-defect (scalar-scalar) correlator $\langle O_1(x)\hat{O}_2(0)\rangle = \frac{b_{O_1\hat{O}_2}}{|x_\perp|^{\Delta_1-\hat{\Delta}_2}|x|^{2\hat{\Delta}_2}}$ can be mapped to

$$\langle\hat{1}|O_1(x)|\hat{O}_2\rangle = \frac{b_{O_1\hat{O}_2}}{|x_\perp|^{\Delta_1-\hat{\Delta}_2}|x|^{2\hat{\Delta}_2}} \tag{1}$$

where $|\hat{1}\rangle$ is the vacuum state of the dCFT.

Next, we apply the Weyl transformation $\tau = R\ln r$ to map the coordinates (r,θ,φ) in $\mathbb{R}^3$ to (τ,θ,φ) in $S^2 \times \mathbb{R}$, where R is the radius of S^2. Under the Weyl transformation, the operator transforms as:

$$\phi(r,\theta,\varphi) \to \phi(\tau,\theta,\varphi) = \Lambda(r,\theta,\varphi)^{\Delta/2}\phi(r,\theta,\varphi), \tag{2}$$

where $\Lambda = R^{-2}e^{\frac{2\tau}{R}}$ represents the scale factor of this transformation. Substituting this into the correlator and setting $\tau = 0$, we get:

$$\langle\hat{1}|O_1(\tau=0,\theta,\varphi)|\hat{O}_2\rangle = \langle\hat{1}|\Lambda(r,\theta,\varphi)^{\Delta_1/2}O_1(r,\theta,\varphi)|\hat{O}_2\rangle\big|_{r=1} = R^{-\Delta_1}\frac{b_{O_1\hat{O}_2}}{|\sin\theta|^{\Delta_1-\hat{\Delta}_2}} \tag{3}$$

On the hand, we have $\langle 1|O_1(\tau=0,\theta,\varphi)|O_1\rangle = R^{-\Delta_1}$ for the bulk CFT, so we finally have,

$$G_{O_1\hat{O}_2} \equiv \frac{\langle\hat{1}|O_1(\tau=0,\theta,\varphi)|\hat{O}_2\rangle}{\langle 1|O_1(\tau=0,\theta,\varphi)|O_1\rangle} = \frac{b_{O_1\hat{O}_2}}{(\sin\theta)^{\Delta_1-\hat{\Delta}_2}} \tag{4}$$

Lastly, it is noting that, for the flat line defect ($d = 1$) breaking the symmetry into $SO(2,1) \times SO(2)$, the defect operator can have a non-trivial $SO(2)$ quantum number $L_z = m$. For such a defect operator, we have,

$$G_{O_1\hat{O}_2} \equiv \frac{e^{-im\varphi}\langle\hat{1}|O_1(\tau=0,\theta,\varphi)|\hat{O}_2, L_z=m\rangle}{\langle 1|O_1(\tau=0,\theta,\varphi)|O_1\rangle} = \frac{b_{O_1\hat{O}_2}}{(\sin\theta)^{\Delta_1-\hat{\Delta}_2}}, \tag{5}$$

where the bulk operator O_1 is still a Lorentz scalar.

Supplementary Note 2: Error analysis

Scaling dimension

In this section, we provide a detailed analysis of the scaling dimensions of primaries and offer a way to estimate the error of the obtained numerical data. In general, in this work, we use two different methods to obtain the scaling dimensions of the defect primaries, which gives consistent results.

These three methods are described as below.

1. Since the scaling dimension of the displacement operator is expected to be $\Delta_{\hat{D}} = 2$, we can set the dimension of the displacement operator to $\Delta_{\hat{D}} = 2$ and rescale the energy spectrum. The obtained results of low-lying defect primaries are shown in the first line of Supplementary Table I.

2. We assume that the presence of defect does not affect the velocity of spectra, so we let $v_{\text{def}} = v_{\text{bulk}}$. So we determine the scaling dimensions $\Delta_{\hat{O}}$ through

$$E_{\hat{O}} - E_{\hat{1}} = \frac{\Delta_{\hat{O}}}{R} v_{\text{bulk}},$$

where v_{bulk} is determined by the bulk Ising CFT [1]. That is, after extracting the velocity v_{bulk}, the scaling dimensions of the dCFT is

$$\Delta_{\hat{O}} = \frac{E_{\hat{O}} - E_{\hat{1}}}{v_{\text{bulk}}} R = \frac{E_{\hat{O}} - E_{\hat{1}}}{E_\sigma - E_0} \Delta_\sigma,$$

where $\Delta_\sigma = 0.518149$ is for bulk primary σ field. The results obtained in this way are displayed in the second line of Supplementary Table I. The consistency of methods 1 and 2 is strong evidence of $v_{\text{def}} = v_{\text{bulk}}$. Throughout this article, we employ this method to compute the scaling dimensions.

Supplementary Table I. The scaling dimensions of primaries in sector $L_z = 0, 1$. The first two lines correspond to method 1-2 in size $N = 36$.

	$L_z = 0$			$L_z = 1$		
	$\hat{\phi}$	$\hat{\phi}'$	$\hat{\phi}''$	$\hat{D}$	$\hat{\phi}_1$	$\hat{\phi}'_1$
$\Delta_{\hat{O}}^{\text{Method 1}}(N=36)$	1.59	3.05	4.06	2	3.55	4.52
$\Delta_{\hat{O}}^{\text{Method 2}}(N=36)$	1.57	3.02	4.02	1.98	3.51	4.53

The summary of the scaling dimensions obtained by the above two methods is shown in Supplementary Table I.

Furthermore, to estimate the scaling dimensions in the thermodynamic limit, we apply a finite-size extrapolation analysis based on method 2. Generally, we fit the scaling dimensions of primaries (obtained on finite-size N) using the following form (see section C):

$$\Delta_{\hat{O}}^{\text{Method 2}}(N) \approx \Delta_{\hat{O}} + \frac{b}{R^{\Delta_{\hat{\phi}}-1}} + \frac{c}{R^{\Delta_{\hat{\phi}'}-1}} + \text{higher order corrections} \tag{6}$$

where b, c are non-universal parameters, $R \sim \sqrt{N}$. The finite-size scaling for several typical primaries are shown in Supplementary Figure 2. The scaling dimension in the thermodynamic limit $\Delta_{\hat{O}}$ can be extracted in this way.

At last, the relative error is estimated through the following comparison. The numerical error of the fitting process in Supplementary Eq. (6) is $\delta\overline{\Delta}_{\hat{O}}$, which is determined by comparing the different fitting processes using various finite system sizes (five large system sizes are always included). We also compare different methods to determine the relative errors, e.g. difference between $\Delta_{\hat{O}}$ and $\Delta_{\hat{O}}^{\text{Method 1}}(N = 36), \Delta_{\hat{O}}^{\text{Method 2}}(N = 36)$ as the relative error. Finally, we use the maximum value of these estimates as the relative error:

$$\delta\Delta_{\hat{O}} = Max\{\delta\overline{\Delta}_{\hat{O}}, |\Delta_{\hat{O}} - \Delta_{\hat{O}}^{\text{Method 1}}(N = 36)|, |\Delta_{\hat{O}} - \Delta_{\hat{O}}^{\text{Method 2}}(N = 36)|\}. \tag{7}$$

We think this error represents the maximal relative error of the obtained scaling dimensionsin our numerical calculations.

Finally, the numerical estimation based on the finite-size extrapolation and corresponding error bars are presented in the Table I in the main text.

OPE coefficients

In this subsection, we delve into the finite size correction and error estimation of the OPE coefficients. Generally, the finite size correction arises from the descendants and higher primary operators. For instance, considering σ, the higher contributions involve $\partial_\mu\sigma$, $(\Box\sigma, \partial_\mu\partial_\nu\sigma)$, $(\partial_\mu\Box\sigma, \partial_\mu\partial_\nu\partial_\rho\sigma)$, σ', and so on. The corresponding bulk-defect OPE is given by:

$$G_{\sigma\hat{O}}(\theta) = \frac{\langle\hat{1}|n^z|\hat{O}\rangle}{\langle\sigma|n^z|1\rangle} \approx \frac{b_{\sigma\hat{O}}}{(\sin\theta)^{\Delta_\sigma-\Delta_{\hat{O}}}}\left(1+\frac{c}{R}+\frac{c'}{R^2}+\frac{c''}{R^3}+O(1/R^{4.180-0.518})\right). \quad (8)$$

Similarly, for the higher correction from $\partial_\mu\epsilon$, $T_{\mu\nu}$, $(\Box\epsilon, \partial_\mu\partial_\nu\epsilon)$, ϵ', and so on, we have:

$$G_{\epsilon\hat{O}}(\theta) = \frac{\langle\hat{1}|n^x|\hat{O}\rangle - \delta_{\hat{O}\hat{1}}\langle\hat{1}|n^x|\hat{1}\rangle}{\langle\epsilon|n^x|1\rangle} \approx \frac{b_{\epsilon\hat{O}}}{(\sin\theta)^{\Delta_\epsilon-\Delta_{\hat{O}}}}\left(1+\frac{c}{R}+\frac{c'}{R^{3-1.413}}+\frac{c''}{R^2}+O(1/R^{3.830-1.413})\right). \quad (9)$$

Regarding the error bar estimation, we perform the finite size extrapolation using the first two powers to obtain the OPE coefficients $b_{O\hat{O}_n}$. We compare various fitting processes by considering different finite system sizes used in the finite-size extrapolations (the five largest system sizes are always included). By comparing the extrapolated OPE coefficients obtained by different fitting processes, we calculate the standard value and corresponding error bar.

Supplementary Note 3: Attractive fixed point

In this section, we aim to demonstrate numerically that the Ising CFT with a line defect possesses an attractive fixed point under the flow of h_d.

Firstly, we ensure that, under the magnetic line defect the low-energy excitation spectrum is gapless. This can be examined through the scaling analysis, as illustrated in Supplementary Figure 1.

Second, we examine how the scaling dimensions of primaries converge to the same values for various h_d. Finite size corrections arise from irrelevant operators with scaling dimensions $\Delta_{\hat{O}} > 1$. Among these operators, the lowest two primaries are $\hat{\phi}$ and $\hat{\phi}'$. Consequently, the finite size correction to the scaling dimension can be approximated as follows up to the first order perturbation:

$$\Delta_{\hat{O}}(N) \approx \Delta_{\hat{O}} + \frac{b}{R^{\Delta_{\hat{\phi}}-1}} + \frac{c}{R^{\Delta_{\hat{\phi}'}-1}} + \text{higher order corrections}. \quad (10)$$

By employing this relation and setting $\hat{O} = \hat{\phi}, \hat{\phi}'$, we obtain two consistency equations which give the scaling dimensions $\Delta_{\hat{\phi}}, \Delta_{\hat{\phi}'}$. For example, we obtain for $h_d = 300$:

$$\Delta_{\hat{\phi}} \approx 1.63 \quad \text{and} \quad \Delta_{\hat{\phi}'} \approx 3.12. \tag{11}$$

One may wonder how the second-order perturbation influences the extrapolated data. We also try to do the finite-size extrapolation using the scaling function by involving the leading second order perturbation

$$\Delta_{\hat{O}}(N) \approx \Delta_{\hat{O}} + \frac{b}{R^{\Delta_{\hat{\phi}}-1}} + \frac{c}{R^{2(\Delta_{\hat{\phi}}-1)}} + \text{higher order corrections.} \tag{12}$$

Using Supplementary Supplementary Eq. (12), we obtain for $h_d = 300$:

$$\Delta_{\hat{\phi}} \approx 1.62 \quad \text{and} \quad \Delta_{\hat{\phi}'} \approx 3.08, \tag{13}$$

which is almost the same as those obtained in Supplementary Eq. (11). So we conclude that higher-order corrections do not change the extrapolated scaling dimensions qualitatively.

Subsequently, by applying this approach to various values of h_d ranging from 1 to 1000, we observe that the scaling dimensions of $\hat{\phi}$ and $\hat{\phi}'$ are insensitive to h_d. We examine the displacement operator $\hat{D}$ in the $L_z = 1$ sector. The exact value is $\Delta_{\hat{D}} = 2$, and the numerical results closely match 2 with very high precision. These findings, the scaling dimensions insensitive to the defect strength h_d, provide compelling evidence for the existence of an attractive fixed point in the presence of a line defect. All the results are presented in Supplementary Figure 2.

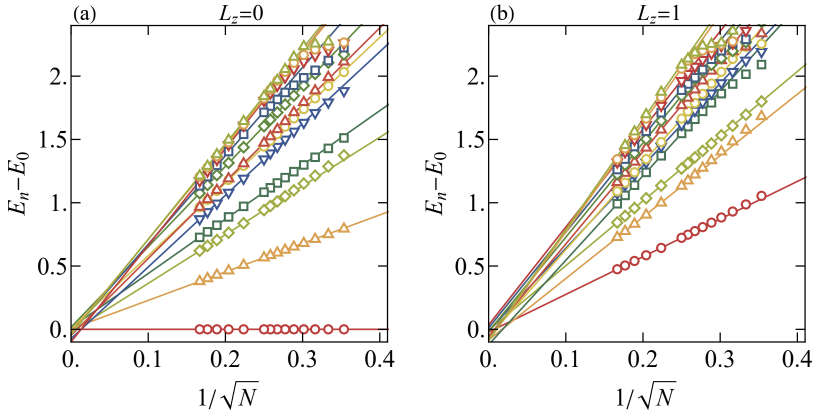

Supplementary Figure 1. **Energy gap scaling.** The finite-size scaling of energy gap $E_n - E_0$ for the first 10 states in sector (a)$L_z = 0$ and (b)$L_z = 1$. System sizes from $N = 8$ to $N = 15$ (ED) and $N = 16$ to $N = 36$ (DMRG with $D = 5000$). Here we set $h_d = 300$.

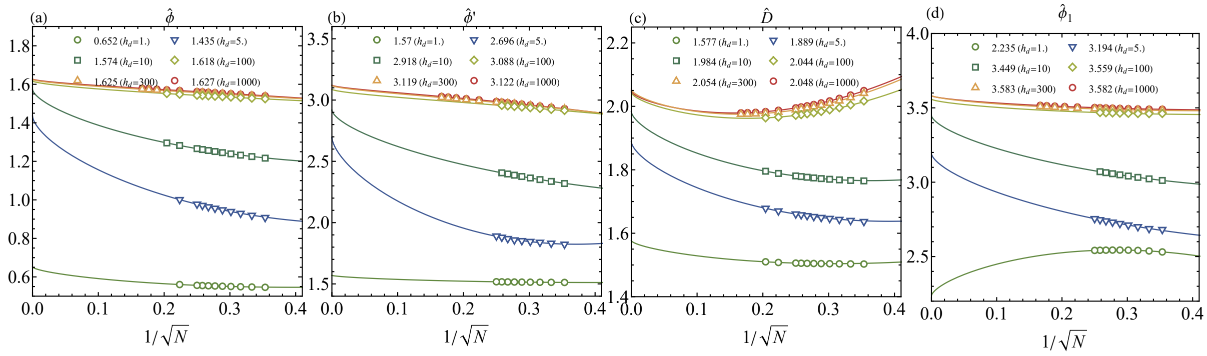

Supplementary Figure 2. **Finite-size correction of scaling dimensions** for the lower primaries: (a) $\hat{\phi}$, (b) $\hat{\phi}'$, (c) $\hat{D}$, and (d) $\hat{\phi}_1$, are determined using Supplementary Eq. (10). For different values of h_d, each primary converges to the same value, indicating the nature of an attractive fixed point.

Finally, we can further consider the limit of $h_d \to \infty$, as shown in Sec. F. In this limit, we demonstrate that the whole spectra is almost the same with the large h_d regime. This indicates that the attractive fixed point is realized in the large h_d limit.

Supplementary Note 4: Numerical results of correlators of ϵ

In the main text, we have shown the correlators and OPE coefficients related to σ (Fig. 4 and related discussion). In this section, we present the correlators and OPE coefficients related to the

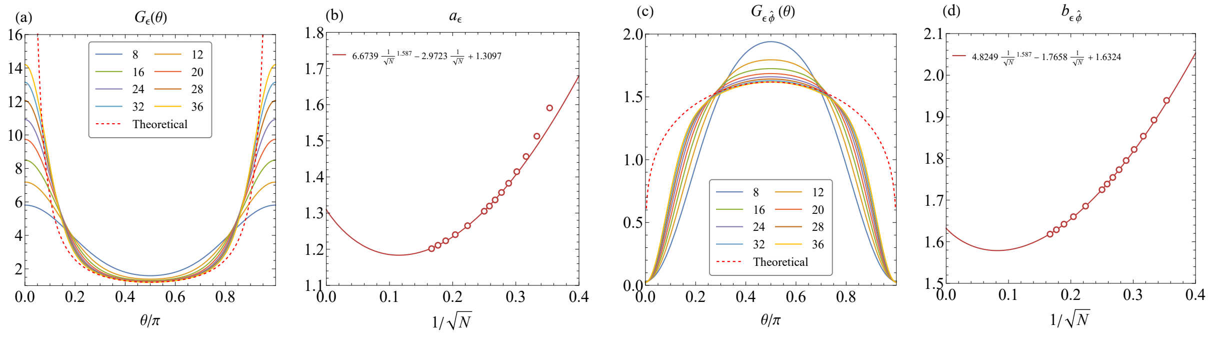

Supplementary Figure 3. **Correlators related to** ϵ (a-b) The one-point correlator a_ϵ and its finite-size extrapolation. The result is $a_\epsilon \approx 1.31$. (c-d) The bulk-defect correlator $G_{\epsilon\hat{\phi}}(\theta)$ with a resulting coefficient of $b_{\epsilon\hat{\phi}} \approx 1.63$. The dashed lines correspond to the theoretical correlator in Supplementary Eq. (14) with $b_{\epsilon\hat{O}}$ and $\Delta_{\hat{O}}$ from the $N = 36$ curves. The system size ranges from $N = 8$ to $N = 36 (D = 5000)$.

bulk operator ϵ. Following the method described in the main text and in Supplementary References [2], we approximate it using the operator $n^x(\Omega)$. The one-point and bulk-defect OPE are given by:

$$\begin{aligned} G_\epsilon(\theta) &= \frac{\langle \hat{1}|n^x|\hat{1}\rangle - \langle 1|n^x|1\rangle}{\langle \epsilon|n^x|1\rangle} \approx \frac{a_\epsilon}{(\sin\theta)^{\Delta_\epsilon}} + O(1/R) \\ G_{\epsilon\hat{O}}(\theta) &= \frac{\langle \hat{1}|n^x|\hat{O}\rangle}{\langle \epsilon|n^x|1\rangle} \approx \frac{b_{\epsilon\hat{O}}}{(\sin\theta)^{\Delta_\epsilon - \Delta_{\hat{O}}}} + O(1/R) \end{aligned} \tag{14}$$

Please note that the operator n^x includes an identity component that should be subtracted. Since $\Delta_\epsilon \approx 1.413 > 0$, $G_\epsilon(\theta)$ should diverge at $\theta = 0, \pi$. This behavior is also observed in Supplementary Figure 3(a). We calculated $G_\epsilon(\theta)$ and extracted the OPE coefficient using $a_\epsilon = G_\epsilon(\pi/2)$. To perform the finite-size extrapolation, we need to consider the finite-size correction given by:

$$G_\epsilon(\theta) \approx \frac{1}{(\sin\theta)^{\Delta_\epsilon - \Delta_{\hat{O}}}} \left(b_{\epsilon\hat{O}} + \frac{b_{\partial\epsilon\hat{O}}}{R} + \frac{b_{T_{\mu\nu}\hat{O}}}{R^{3-1.413}} + O(1/R^2) \right) \tag{15}$$

where the first pole $1/R$ comes from $\partial\epsilon$ and the second pole comes from $T_{\mu\nu}$. After the finite-size extrapolation, we find $a_\epsilon \approx 1.31$.

Next, we consider the bulk-defect OPE. Since the lowest primary in dCFT is $\Delta_{\hat{\phi}} \approx 1.64 > \Delta_\epsilon$, the correlator $G_{\epsilon\hat{O}}(\theta)$ is zero at $\theta = 0, \pi$. We present the result for $G_{\epsilon\hat{\phi}}$ in Supplementary Figure 3(c), and the finite-size correction follows the same analysis as described above, yielding $b_{\epsilon\hat{\phi}} \approx 1.63$.

Supplementary Note 5: Ward identity regarding to the displacement operator

In dCFT, the displacement operator is related to the stress tensor in the bulk CFT, $\partial_\mu T_{\mu\nu}(x_\perp, x_\parallel) = \delta(x_\perp)\hat{D}_\nu(x_\parallel)$. The stress-tensor has a canonical normalization through the Ward-identity, so the normalization of $\hat{D}$ is also fixed. Therefore, the two-point correlator of displacement operator will not be normalized to 1, instead, it is

$$\langle \hat{D}_i(x)\hat{D}_j(0)\rangle = C_{\hat{D}} \frac{\delta_{ij}}{x^4}, \tag{16}$$

where the normalization factor $C_{\hat{D}}$ is the Zamolodchikov norm [3] or central charge. For this canonically normalized displacement operator, its bulk-defect OPE coefficients are constrained by the Ward identity [3]:

$$\Delta_O a_O = \frac{\pi}{2} b_{O\hat{D}}, \tag{17}$$

where O is a bulk scalar primary operator. $b_{O\hat{D}}$ is a bulk-defect OPE coefficient defined by the two-point correlator in $\mathbb{R}^3$,

$$\langle O(x)\hat{D}_i(0)\rangle = b_{O\hat{D}}\frac{x_{\perp,i}}{|x_\perp|^{\Delta-1}|x|^4}. \tag{18}$$

In our fuzzy sphere computation, we can directly use the state of displacement operator to compute the correlators involving the displacement operator. Specifically, we have state with well defined $SO(2)\cong U(1)$ quantum number L_z,

$$|\hat{D}, L_z=\pm 1\rangle = \frac{1}{\sqrt{2C_{\hat{D}}}}(\hat{D}_1(\tau=-\infty)\pm i\hat{D}_2(\tau=-\infty))|\hat{1}\rangle. \tag{19}$$

The numerical factor $1/\sqrt{2C_{\hat{D}}}$ is chosen such that $\langle\hat{D}, L_z = 1|\hat{D}, L_z = 1\rangle = \langle\hat{D}, L_z = -1|\hat{D}, L_z = -1\rangle = 1$. On the cylinder $S^2\times\mathbb{R}$, we have

$$\langle\hat{1}|O(\tau=0,\theta,\varphi)|\hat{D}, L_z=1\rangle = \frac{b_{O\hat{D}}}{\sqrt{2C_{\hat{D}}}}\frac{e^{i\varphi}}{R^{\Delta}(\sin\theta)^{\Delta-1}} \tag{20}$$

Therefore, we can extract $C_{\hat{D}}$ using

$$\sqrt{2C_{\hat{D}}} = \frac{2}{\pi}\frac{\Delta_O G_O(\theta=\pi/2)}{G_{O\hat{D}}(\theta=\pi/2)}, \tag{21}$$

where,

$$G_{O\hat{D}} \equiv \frac{e^{-i\varphi}\langle\hat{1}|O(\tau=0,\theta,\varphi)|\hat{D}, L_z=1\rangle}{\langle 1|O(\tau=0,\theta,\varphi)|O\rangle}, \tag{22}$$

The results are depicted in Supplementary Figure 4(c). For comparison, we calculate the results by setting $O=\sigma$ and $O=\epsilon$, yielding $C_{\hat{D}}=0.27(1)$ for $O=\sigma$ and $C_{\hat{D}}=0.30(8)$ for $O=\epsilon$.

Supplementary Note 6: In the limit of $h_d\to\infty$

In this section, we will consider whether the system remains at the dCFT fixed point when the defect strength h_d tends to infinity. First, we consider the defect term

$$\begin{aligned} H_d &= 2\pi h_d\left[n^z(\theta=0,\varphi=0)+n^z(\theta=\pi,\varphi=0)\right] \\ &= 2\pi\sum_l\left[Y_{l,0}(\theta=0,\varphi=0)+Y_{l,0}(\theta=\pi,\varphi=0)\right]n^z_{l,0} \\ &= \sum_{m_1}\left[C_{m_1}(\theta=0)+C_{m_1}(\theta=\pi)\right]\hat{c}^\dagger_{m_1}\sigma^z\hat{c}_{m_1} \end{aligned} \tag{23}$$

where $C_{m_1}(\theta)$ is

$$C_{m_1}(\theta) = \frac{1}{2}h_d\pi\sum_l(-1)^{3s+m_1+l}P_l(\cos\theta)(2l+1)\begin{pmatrix} s & s & l \\ s & -s & 0\end{pmatrix}\begin{pmatrix} s & s & l \\ m_1 & -m_1 & 0\end{pmatrix} \tag{24}$$

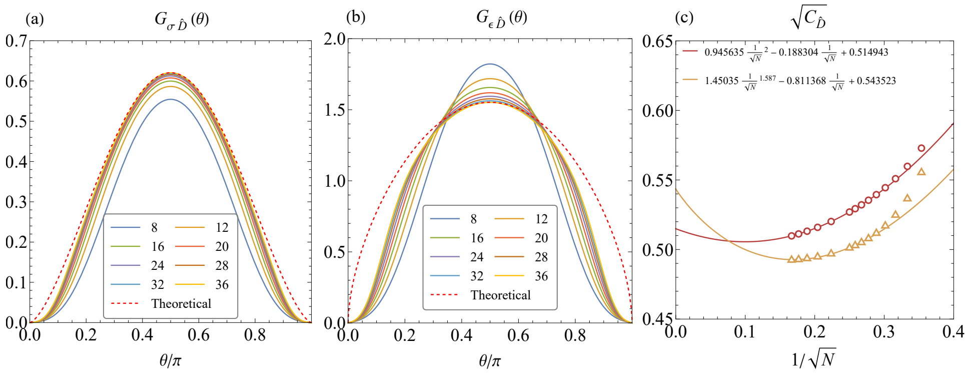

Supplementary Figure 4. Correlators between the displacement operator and bulk operator: (a) $O = \sigma$ and (b) $O = \epsilon$. The dashed lines correspond to the theoretical correlator in Supplementary Eq. (14) with $b_{\epsilon\hat{D}}$ and $\Delta_{\hat{D}}$ from the $N = 36$ curves. (c) The extracted Zamolodchikov norm from Supplementary Eq. (21). The red circles represent the results obtained when $O = \sigma$, while the yellow triangles correspond to the results when $O = \epsilon$.

The Legendre polynomial $P_l(x)$ have special values $P_l(1) = 1$ and $P_l(-1) = (-1)^l$, substituting into the last equation and using the orthogonal relation of $3j-$symbol, we have

$$C_{m_1}(\theta = 0) = \frac{1}{2} h_d \delta_{m_1,-s} \qquad C_{m_1}(\theta = \pi) = \frac{1}{2} h_d \delta_{m_1,s}. \tag{25}$$

Thus, the defect term has a simplified form in orbital space (see also [4])

$$H_d = \frac{1}{2} h_d \left(\hat{c}_s^\dagger \sigma^z \hat{c}_s + \hat{c}_{-s}^\dagger \sigma^z \hat{c}_{-s} \right). \tag{26}$$

In orbital space, the defect term only acts on the $m = \pm s$ orbitals. This implies that if the defect strength h_d is sufficiently large, we can fix the spins of the $m = \pm s$ orbitals to $\downarrow$ in numerical computations and then optimize the other orbitals. This approach brings two advantages: firstly, it reduces the number of orbitals, which can enhance computational efficiency; secondly, it avoids numerical difficulties arising from excessively large h_d.

Using the method described in Supple. Mat. Sec. B, we similarly computed the spectra for N ranging from 8 to 36 at $h_d = \infty$ and plotted them in Supplementary Figure 5. We can observe that the system still exhibits conformal symmetry, indicating it remains at the fixed point of dCFT. Additionally, we list the scaling dimensions of several lower primary fields in Supplementary Table II. A comparison with the finite $h_d = 300$ case mentioned in the main text reveals consistency within the error range. This indicates that the choice of $h_d = 300$ in the main text is sufficiently large. Combining this observation with the discussion in the main text, we can confidently assert

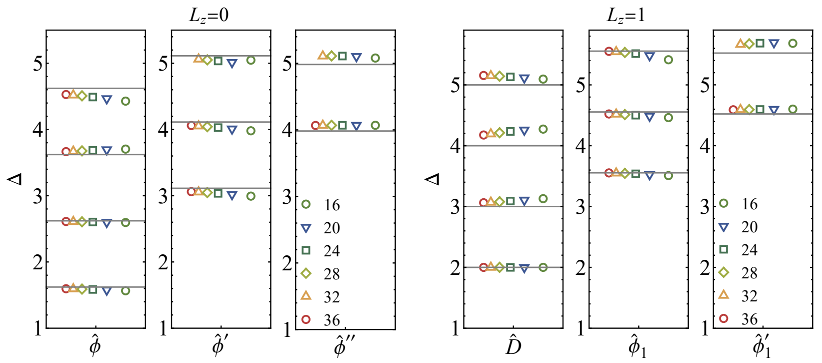

Supplementary Figure 5. Conformal tower of defect primaries in $h_d \to \infty$. Defect primary fields and their descendants with global symmetry (a) $L_z = 0$ and (b) $L_z = 1$. The grey horizon lines stand for the theoretical expectation for descendants. Different colored symbols represent the results based on various system sizes. By increasing system size N all of the scaling dimensions approach the theoretical values consistently, supporting an emergent conformal symmetry in the thermodynamic limit.

Supplementary Table II. The scaling dimensions of primary fields under infinite and finite defect strengths, are determined through the state-operator correspondence on the fuzzy sphere. Please see a detailed analysis of errors and finite-size extrapolation in Supple. Mat. Sec. B-C.

	$L_z = 0$			$L_z = 1$		
	$\hat{\phi}$	$\hat{\phi}'$	$\hat{\phi}''$	$\hat{D}$	$\hat{\phi}_1$	$\hat{\phi}'_1$
$h_d = \infty$	1.63(4)	3.12(9)	4.04(3)	2.05(7)	3.58(7)	4.62(8)
$h_d = 300$	1.63(6)	3.12(10)	4.06(18)	2.05(7)	3.58(7)	4.64(14)

the existence of an attractive dCFT fixed point at $h_d = \infty$ for the 3D Ising critical point under the imposition of the magnetic field line.

* yhe@perimeterinstitute.ca

† zhuwei@westlake.edu.cn

[1] W. Zhu, C. Han, E. Huffman, J. S. Hofmann, and Y.-C. He, Phys. Rev. X **13**, 021009 (2023).

[2] L. Hu, Y.-C. He, and W. Zhu, Phys. Rev. Lett. **131**, 031601 (2023).

[3] M. Billò, V. Gonçalves, E. Lauria, and M. Meineri, Journal of High Energy Physics **2016**, 91 (2016), arXiv:1601.02883 [hep-th].

[4] Z. Zhou, D. Gaiotto, Y.-C. He, and Y. Zou, "The g-function and defect changing operators from wave-function overlap on a fuzzy sphere," (2024), arXiv:2401.00039 [hep-th].
